# Evaluating walkability across age groups and flooring materials using IMU sensors

**DOI:** 10.3389/fpubh.2024.1509602

**Published:** 2024-12-05

**Authors:** Daehwi Jo, Hyunsoo Kim

**Affiliations:** Department of Architectural Engineering, Dankook University, Yongin-si, Gyeonggi-do, Republic of Korea

**Keywords:** walkability, flooring materials, IMU sensors, dynamic time warping, fatigue, age

## Abstract

This study presents a novel approach to quantitatively assess the impact of flooring materials on walkability using Inertial Measurement Unit (IMU) sensors and Dynamic Time Warping (DTW) algorithm. Four common pavement materials (wood, asphalt, concrete block, and cement) were evaluated across five age groups (20–30, 30–40, 40–50, 50–60, and over 60 years) with 80 participants walking 1,200 m on each surface. IMU sensors attached to the lumbar region recorded acceleration and gyroscope data, which were then analyzed using DTW to quantify gait stability. Results showed significant differences in DTW values among materials, with wood exhibiting the lowest average DTW value (12.99 ± 3.05) indicating the most stable walking environment, while cement showed the highest (39.14 ± 9.74). In addition, age-related analysis revealed increasing DTW values with age across all materials, with the most pronounced effect in the older adult group. The methodology presented offers sensor-based approach for evaluating and optimizing pedestrian infrastructure in smart city development.

## Introduction

Increasing physical activity in modern society is a key component of improving public health (1). Among the various physical activities, walking is one of the most basic and universal and can be easily engaged in by people of all ages (2, 3). Walking is associated with a variety of health benefits, including reduced obesity (4, 5), improved cardiovascular health (6, 7), diabetes management (8, 9), and improved quality of life (10, 11). In urban environments, the quality of the pedestrian environment is closely related to the walking activity of citizens, as most walking activity takes place on sidewalks (12). Among the various elements of the walking environment, sidewalk pavement has a direct impact on pedestrians’ safety, comfort, and overall walking experience (13). Each pavement has unique properties, such as stiffness, rebound force, and modulus of elasticity, which affect pedestrians’ walking patterns and comfortness (14). Walking and the ability to walk are heavily influenced by these external conditions. The activity of walking is essentially a direct interaction between the foot and the ground (15). There are many different pavement materials for sidewalks, including asphalt, cement, concrete, and wood, and each has its own characteristics and properties. Therefore, understanding the relationship between pavement materials and walking behavior is an important step in designing and building urban environments that promote physical activity (16, 17). In this context, the relationship between sidewalk characteristics and walkability has been an important research topic in urban planning and public health for decades (18–20). In particular, the impact of pavement materials on the pedestrian experience has been studied continuously (21–23), but these studies have mainly focused on the overall qualitative assessment of sidewalks. On the other hand, there is a relative lack of research on the direct impact of pavement materials on walking patterns.

Recent advances in sensing technologies have enabled objective and quantitative measurements of walkability (24–27), and several studies have utilized them to evaluate walkability and pavement condition. Kim et al. (28) proposed a method to detect sidewalk defects using Inertial Measurement Unit (IMU) and evaluate pavement condition by analyzing changes in pedestrians’ walking patterns. Wang et al. (29) developed a method to evaluate the quality of the walking environment using the accelerometer of a smartphone. However, these studies mainly focus on the physical condition of the sidewalk or the overall quality of the walking environment, and there is a relative lack of research on the direct impact of pavement materials on walking patterns. To overcome these limitations, this study combines IMU sensors with a Dynamic Time Warping (DTW) algorithm to precisely analyze the effects of different pavement materials on walking patterns. The DTW algorithm measures the similarity of time series data, which is useful for comparing walking patterns under different pavement materials. Gait data collection using IMU sensors can provide continuous and objective data (30), and includes accelerometers, gyroscopes, geomagnetometers, etc. to precisely measure pedestrian movements. This allows for accurate analysis of various gait characteristics such as walking speed, stride length, gait cycle, and foot landing patterns (31). The DTW algorithm can be used to effectively analyze non-linear changes in the gait cycle, which allows for a more precise assessment of the impact of different pavement materials on gait (32). For example, by comparing gait patterns on different pavement materials with DTW, it is possible to quantitatively analyze how each material affects the rhythm and stability of gait. Furthermore, by combining these sensing technologies with data analysis methods, it is possible to build a system that can continuously monitor and evaluate the quality of the walking environment (32). This can provide a basis for understanding the condition of the walking environment in real time and quickly identify necessary improvements. Based on this approach, this study quantitatively measures the subtle changes in walking patterns due to different pavement materials, analyzes the differences in the effects of pavement materials on walking among different age groups, and compares the changes in fatigue with walking distance by pavement material. Through this comprehensive approach, the authors aim to gain a deeper understanding of the effects of pavement materials on walkability and contribute to the design of walking environments that consider different age groups and walking conditions.

## Literature review

Walkability is an important measurement tool for assessing the quality of urban environments, and its importance has been emphasized for a variety of reasons, including citizen health, the environment, and social interaction. Hijriyah et al. (33) define walkability as “the degree to which the built environment supports and encourages walking” and suggest five urban design factors that directly influence walkability. Image refers to the overall visual appeal and maintenance that affects pedestrian safety perception, while sense of place relates to the area’s distinctive character that promotes walking activity. The human scale considers the size and arrangement of physical elements that match walking speeds and comfort. Transparency indicates the visibility of activities beyond the street edge, enhancing perceived safety, and complexity describes the variety of elements that maintain pedestrian interest. These interconnected factors collectively create environments that encourage walking by improving both functional and experiential aspects of pedestrian spaces. Forsyth et al. (34) expands on this concept to include not only physical characteristics, but also the socioeconomic characteristics of the community, safety, and accessibility. As one of the key elements of the walking environment, sidewalk pavement materials directly impact safety, comfort, and overall experience. Prior studies indicate that older adults, especially those over 50, may feel more discomfort and instability on harder surfaces like cement (35). Studies have noted that older adults may experience a reduction in walking stability and comfort on hard surfaces due to increased sensitivity to impact and decreased muscle resilience, which highlights the need for age-specific considerations in urban design. Previous studies have examined various pavement materials and their effects. Ferreira et al. (36) evaluated concrete, interlocking blocks, and asphalt pavements, finding that material selection significantly influenced walking speed (ranging from 1.2 to 1.5 m/s in adults aged 20–40, decreasing to 0.8 to 1.2 m/s in those over 60), pedestrian density (1.2 to 1.8 persons/m^2^), and flow patterns. Li et al. (37) focused on three common materials—concrete, asphalt, and stone pavements—and found that surface texture variations affected skid resistance values by 15–30%, directly impacting walking safety with their effects being most pronounced among older adults (aged 60 and above). Ren et al. studied cement, asphalt, and wood surfaces, documenting temperature variations of up to 15°C between materials, affecting pedestrian thermal comfort. Based on these studies and the prevalence of certain materials in urban environments globally, we selected wood, asphalt, concrete block, and cement for our investigation as they represent the most studied materials, offer distinct physical properties affecting walkability, and provide a comprehensive range of surface characteristics. Additionally, wood is flexible with shock-absorbing qualities, while cement is hard and rigid, potentially increasing impact forces during walking. Such material differences can influence pedestrian comfort and stability (38). While Giles-Corti et al. (39) highlighted the importance of overall space characteristics on walking activity, most previous studies relied on subjective assessments, limiting their ability to objectively measure the direct interaction between actual walking behavior and pavement materials. However, these methodologies incorporate a systematic analysis with both quantitative and qualitative measures to minimize bias, ensuring results are grounded in empirical data rather than subjective judgments. This approach establishes a solid foundation for future research and offers reliable insights into further studies.

Recent advances in sensing technology are providing a new approach to walkability assessment (40–42). These technological advances have greatly improved the accuracy and efficiency of gait analysis, and a variety of methodologies have been developed. Wong et al. (43) presented a method for analyzing pedestrian movement using computer vision technology, Bamberg et al. (44) developed a method for analyzing gait patterns using pressure sensors embedded in shoes, and Bernardina et al. (45) performed gait analysis using a high-precision optical motion capture system. However, each of these techniques has limitations. Computer vision techniques, for example, face privacy issues and show reduced accuracy in complex environments, such as areas with obstacles, uneven surfaces, or high pedestrian traffic that can impede data collection accuracy. Pressure sensors need to be embedded in shoes, which limits their practicality for continuous use in daily life, and optical motion capture systems are challenging to use in real-world settings due to their high cost and restricted measurement space. In contrast, our study utilized a research-grade IMU sensor, costing approximately $1,000, chosen for its high accuracy and data quality. However, there are more cost-effective alternatives, such as IMU sensors embedded in smartphones. For instance, Kim et al. (28) successfully used smartphone IMUs to assess sidewalk conditions, showing that lower-cost sensors can still capture adequate data for similar analyzes. An alternative to overcome these limitations is gait analysis using IMUs. IMU sensors have several advantages that make them well suited for walkability assessment (46). IMU sensors can effectively capture human walking patterns with sampling frequencies adequate for gait analysis (100 Hz in our study), comparable to the capabilities of common smartphone accelerometers. This sampling rate is sufficient to detect subtle changes in walking patterns while maintaining data quality. Additionally, their small size and lightweight nature allow for continuous wear during daily activities without impeding natural movement, making them ideal for extended walking studies in real-world environments (47). Building upon these capabilities, they can also collect data in real-world walking environments, rather than in a laboratory setting, can analyze walking patterns, defined as specific gait characteristics like step length, cadence, and foot placement, in real-world situations (48). Since it does not collect video data, it is free from privacy issues, and various indicators such as acceleration and angular velocity can be measured simultaneously, allowing for a comprehensive analysis of gait. Hutabarat et al. (49) comprehensively reviewed gait analysis methods using IMU sensors and emphasized that this technology provides high accuracy and convenience for gait analysis. Yang and Hsu (50) provide a comprehensive review of physical activity monitoring using accelerometer-based wearable motion detectors, explaining that this technology enables quantitative measurements of walking speed, stride length, and gait cycle. Another advantage of IMU sensors is their applicability in a variety of environments. Urban environments are complex and composed of various elements, such as surface materials, pedestrian density, and obstacles, which directly impact walking stability and comfort. These aspects will be the focus of subsequent research to provide a more comprehensive understanding of urban walkability. IMU sensors can be used indoors and outdoors and can collect reliable data in a variety of terrains and weather conditions. This means they can provide more comprehensive and realistic gait data. IMU sensors can also be worn for long periods of time, allowing you to observe changes in walking patterns over time. This can be very useful for analyzing the impact of certain pavement materials on pedestrian fatigue. Due to these advantages of IMU sensors, this study utilizes IMU sensors to evaluate walkability. This allows us to objectively and quantitatively analyze the impact of different pavement materials on walkability in a real-world walking environment. In particular, the data collected by IMU sensors can detect even the slightest changes in walking patterns, allowing for a more precise analysis of the effects of pavement properties on walking. This is the basis for providing information to create more walkable environments.

Based on this background, this study aims to objectively and quantitatively evaluate the effects of various pavement materials on walkability by utilizing IMU sensors and DTW algorithms.

## Methodology

### Research framework

**Figure 1:**
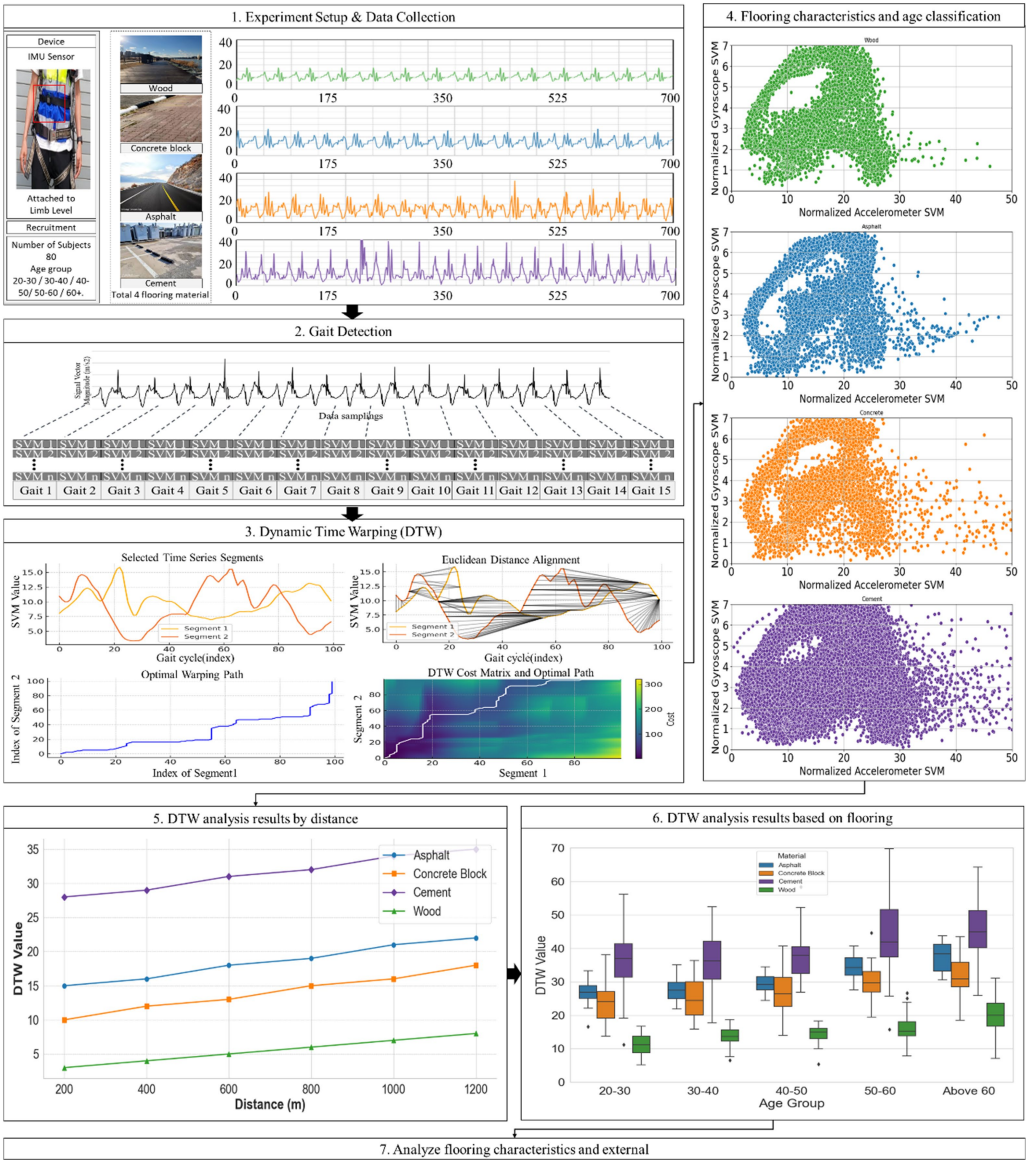
Research framework.

This study adopts a comprehensive approach to evaluate the walkability of different age groups and floor materials by utilizing IMU sensors and DTW analysis technology. The overall research framework consists of six steps: experimental setup and data collection, Gait detection, DTW analysis, flooring characteristics and age classification, DTW analysis results by distance and DTW analysis by age. This framework is schematized in Figure 1.

### Experiment setup

**Figure 2:**
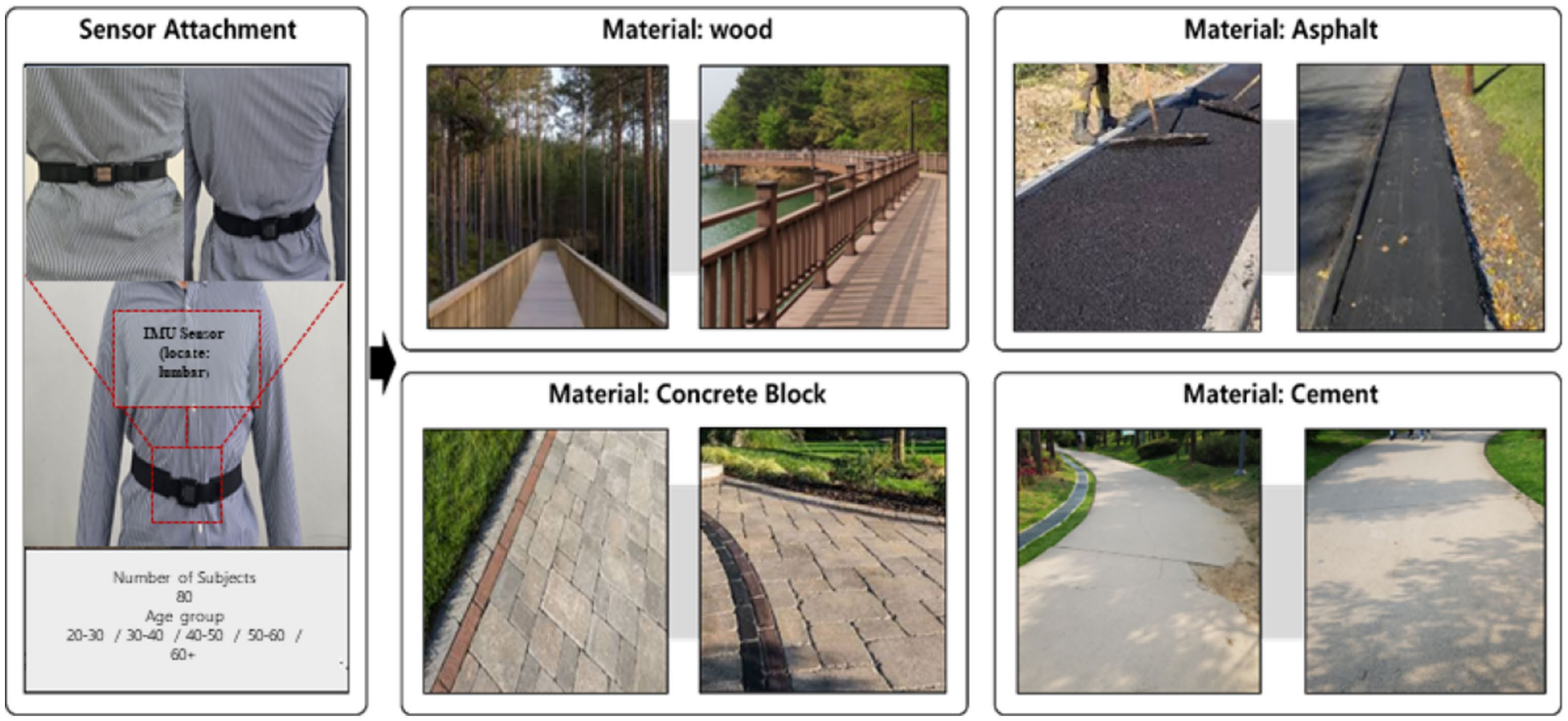
IMU sensor attachment and four walking surfaces.

A total of 80 healthy participants were recruited, with 16 individuals evenly distributed across five age groups: 20–30, 30–40, 40–50, 50–60, and 60+ years. Participants were equipped with a wearable IMU sensor attached to the lumbar region, which corresponds to the body’s Center of Mass (COM). As demonstrated in previous research (51), this location provides the optimal position for capturing whole-body movement and stability during walking with minimal sensor deviation. To evaluate walking patterns across different surfaces, four common sidewalk pavement materials were selected for the study: wood, concrete block, asphalt, and cement. Each material was installed in a controlled environment with a consistent surface area 1,200 m long and at least two meters wide. Participants walked 1,200 m on each flooring material at a comfortable pace of their choice. This distance was determined through preliminary testing which showed that participants began to experience noticeable fatigue after walking approximately 1,000–1,200 m, making it suitable for analyzing changes in walking patterns related to both material properties and accumulated fatigue. The selected distance allows sufficient data collection while avoiding excessive physical strain on participants. A two-day rest period was provided between each walking trial to prevent fatigue accumulation, and the order of flooring materials was randomized for each participant to minimize the influence of previous trials. Figure 2 shows the IMU sensor attachment locations and the four walking paths used in the experiment. The left image shows the location of the IMU sensor attached to the participant’s waist, while the right images show the four different walking surface materials used in the experiment: wood, asphalt, concrete block, and cement.

### IMU data preprocessing and gait cycle segmentation

**Figure 3:**
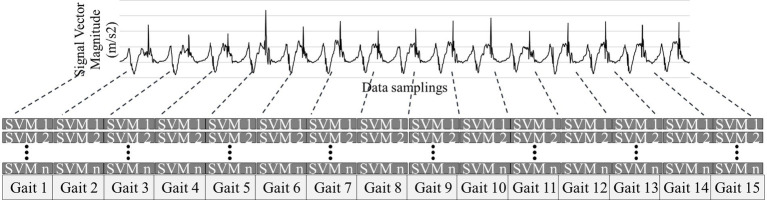
Preprocessing of IMU data using signal vector magnitude (SVM) filter for gait cycle segmentation.

Preprocessing of the raw IMU data is performed using a Signal Vector Magnitude (SVM) filter. This process removes the high-intensity noise contained in the raw signal input to the system and converts multi-axis acceleration data (ax, ay, az) into a single scalar value using Signal Vector Magnitude (SVM). The SVM is calculated as √ (ax^2^ + ay^2^ + az^2^), where ax, ay, and az represent the acceleration values from the three orthogonal axes (X, Y, Z) of the IMU sensor. This conversion combines the tri-axial acceleration data into a comprehensive single value that represents the overall magnitude of movement, making it easier to analyze overall walking patterns. This stepwise filtering process further refines and analyzes the raw data. The filtering removes high-intensity noise to produce a smooth waveform that highlights the key characteristics of the signal, which is then segmented into individual gait cycles (Gait 1, Gait 2, etc.). The start and end of each cycle is identified, and the preprocessed data is output in a clean form that can be saved or passed on to the next step. Figure 3 shows acceleration data preprocessed using SVM.

### Dynamic time warping analysis

**Figure 4:**
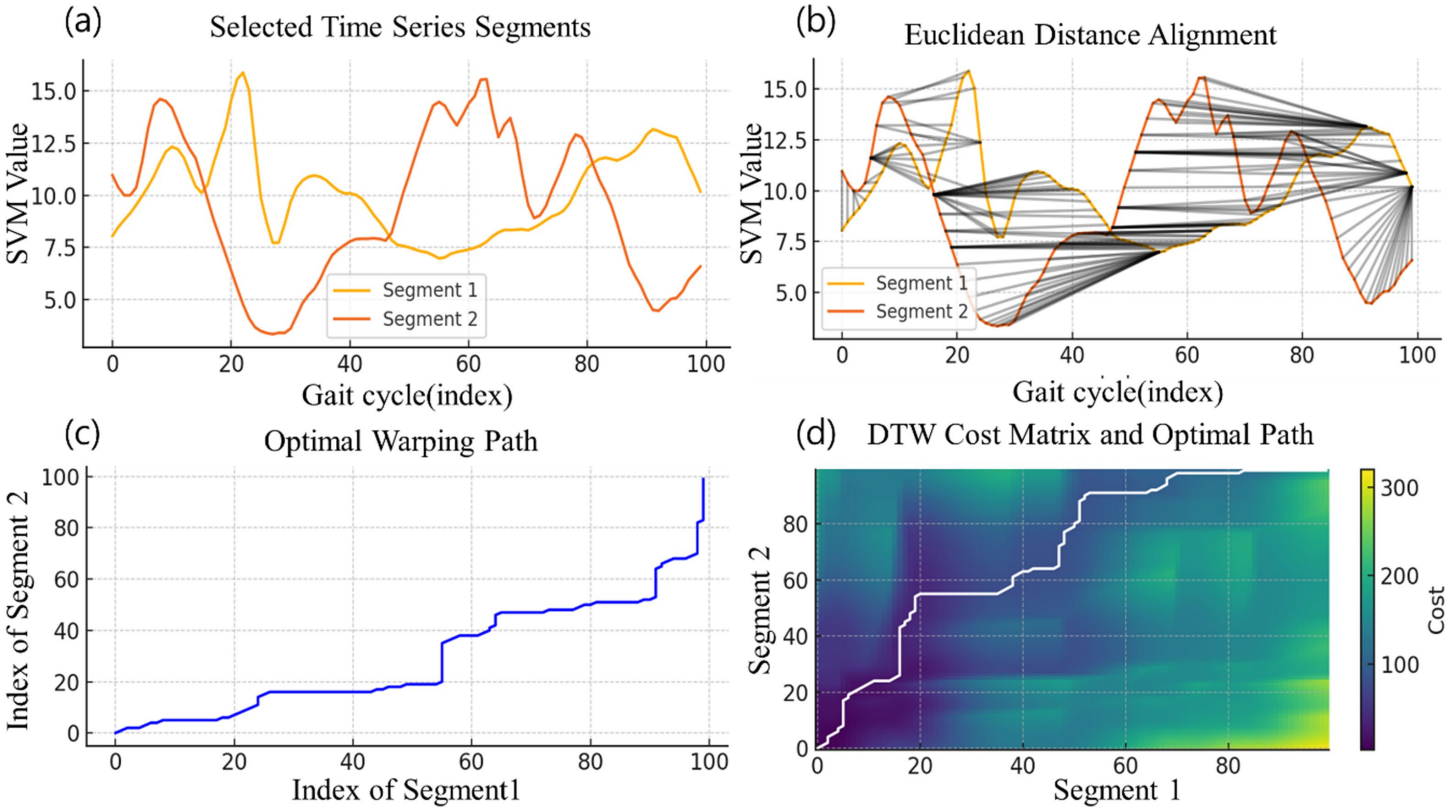
DTW process for comparing gait patterns, **(a)** Select Time Series Segment, **(b)** Euclidean Distance Alignment, **(c)** Optimal Warping Path, **(d)** DTW Cost Matrix and Optimal Path.

DTW analysis is used in this study to quantify the similarity of gait patterns between different flooring materials and age groups. Figure 4 visualizes the DTW analysis process. The analysis process consists of the following steps: First, a selection is made based on the typical walking cycle of each age group on each flooring surface. This is represented by “Segment 1” in Figure 4a. Next, extract the time series data for each gait cycle for all participants and flooring materials. This corresponds to “Segment 2” in Figure 4a, which shows the gait patterns under varying conditions, including four different flooring materials (wood, asphalt, concrete block, and cement), age groups. Compute the DTW distance between the extracted gait cycles and the baseline gait pattern. This process is shown in Figures 4b–d. Figure 4b shows the initial Euclidean distance between two segments as a gray line, which is the starting point for the DTW algorithm. Figure 4c shows the optimal warping path found by the DTW algorithm. This path represents the best way to align the two time series data and compensates for temporal differences in walking patterns. Figure 4d shows the DTW cost matrix and the optimal path at the same time. The color of the heatmap represents the alignment cost at each point, and the white line is the optimal alignment path that was finally selected. The distance calculated along this path becomes the DTW distance between the two walking patterns. Finally, the author calculates the average DTW value per participant for each floor material.

### Comprehensive DTW analysis

The methodology of this study centers on DTW analysis to comprehensively investigate the changes in walking stability with different floor materials, walking distance, and age. Clustering techniques are used to visualize and classify the characteristics of four different flooring materials (wood, concrete block, asphalt, and cement), which are then used to perform DTW analysis based on distance and material classification and age classification. In the DTW analysis by distance and flooring material, the 1,200 m distance is divided into 200 m increments to calculate the average DTW value for six sections. This analysis shows how the walking pattern changes on each flooring material as the walking distance increases.

The DTW by Age analysis compares DTW values for each age group using histograms and boxplots. The histograms provide a visual representation of the frequency distribution of DTW values for each age group, which can be used to characterize the gait patterns of each age group. For example, if the distribution of DTW values in a particular age group is widely spread, this indicates a high variability of gait patterns within that group. On the other hand, if the distribution is narrow and pointed, it indicates that the gait pattern is constant for that group. Boxplots compare the median, interquartile range, and outliers of the DTW value distribution for each age group. This allows you to analyze the overall difference in DTW values between age groups and the nature of the distribution.

The association between DTW values and accumulated walking fatigue is shown in the analysis of study (51). The tendency of DTW values to increase with distance indicates that the stability of the walking pattern decreases as walking fatigue accumulates. If the DTW value increases sharply with distance for a particular material, this indicates that the material may cause fatigue more rapidly over longer distances and compromise gait stability. On the other hand, a material with a DTW value that remains nearly constant may be able to maintain a stable walking pattern over long distances, indicating that fatigue buildup may be relatively low. This analysis can also reveal differences in fatigue buildup based on the characteristics of different flooring materials. For example, when examining DTW value increases across age groups, cement showed the highest rate of increase in the older adult group (60+ years), with DTW values rising from 37.58 to 41.50, indicating rapid fatigue accumulation. In contrast, wood demonstrated more gradual increases across all age groups, with the 20–30 age group showing minimal changes (from 11.82 to 13.25) and even the 60+ age group maintaining relatively stable values (from 11.82 to 15.50). These age-specific responses to different flooring materials indicate that material selection becomes increasingly critical for older pedestrians who are more susceptible to fatigue.

## Results

### Classifying flooring material properties

**Figure 5:**
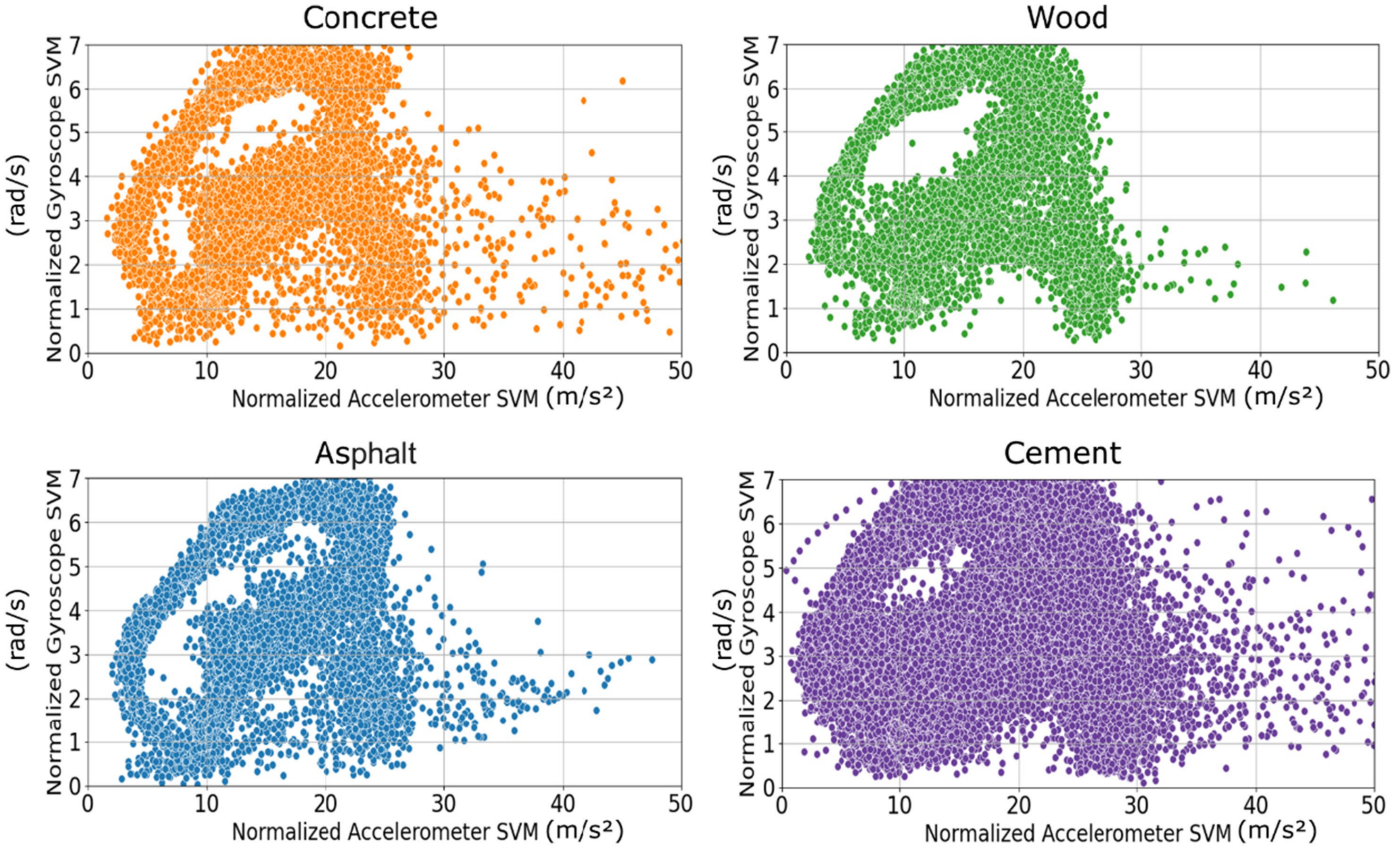
Comparison of accelerometer and gyroscope SVM distributions across different floor materials (concrete, wood, asphalt, cement).

Normalized accelerometer and gyroscope signal vector magnitude (SVM) data collected by IMU sensors were used to analyze gait characteristics for four different floor materials (concrete, wood, asphalt, and cement). From there, a clustering analysis was performed to identify differences in gait patterns that occurred on each floor material. Figure 5 shows the clustering graph of these measures for each floor material.

The analysis revealed distinct patterns for each floor material. For concrete, accelerometer SVM values ranged from 0 to 47.8 m/s, with a mean of 18.3 (SD = 9.2 m/s). The gyroscope SVM values were clustered between 0 and 6.9 m/s, with a mean of 2.8 (SD = 1.4 m/s). The wide distribution of accelerometer values indicates a variety of acceleration patterns, while the more concentrated gyroscope values indicate relatively stable rotational motion during walking. Wood had more concentrated accelerometer SVM values ranging from 0 to 30.5 m/s, with a mean of 14.2 m/s (SD = 6.5 m/s). The gyroscope SVM values ranged from 0 to 6.8 m/s, with a mean of 2.6 m/s (SD = 1.3 m/s). These results show that the elastic properties of wood absorb shocks during walking, creating a more stable and consistent walking pattern. Asphalt had the most concentrated distribution of accelerometer SVM values, ranging from 0 to 34.2 m/s, with a mean of 15.7 m/s (SD = 7.1 m/s). Gyroscope SVM values ranged from 0 to 6.7 m/s, with a mean of 2.5 (SD = 1.2 m/s). The low accelerometer values indicate that asphalt is a more cushioned surface for walking compared to harder materials like concrete or cement. Cement had the widest distribution of all materials, with accelerometer SVM values ranging from 0 to 48.2 m/s, with a mean of 19.1 m/s (SD = 9.5 m/s). Gyroscope SVM values ranged from 0 to 6.8 m/s, with a mean of 2.9 m/s (SD = 1.5 m/s). The distribution pattern was clear, but the mean and standard deviation were slightly higher for both accelerometer and gyroscope measurements. This suggests that cement surfaces, like concrete, cause high impact during walking and result in unstable walking patterns.

Statistical analysis using a one-way analysis of variance ANOVA showed a significant difference in accelerometer SVM values between the four materials, *p* < 0.001. ANOVA analysis also showed a significant difference for the gyroscope SVM values, *p* < 0.001. Table 1 shows the results of the ANOVA analysis. These results show that each flooring material has a unique impact on walking patterns. In particular, the clear difference between hard surfaces (concrete, cement) and relatively soft surfaces (wood, asphalt) indicates that the physical properties of the flooring material have a direct impact on gait dynamics.

**Table 1:** ANOVA results of IMU sensor data for different flooring materials.

Classification		Sum of squares	Degrees of freedom	Mean square	F-ratio	*p*-value
Acceleration	Between	8524.6	3	2841.5	42.8	<0.001
SVM	Within	5043.2	76	66.4		
Value	Total	13567.8	79			
Acceleration	Between	642.3	3	231.1	38.2	<0.001
SVM	Within	425.8	76	5.6		
Value	Total	1068.1	79			

### Analyze cumulative fatigue and gait characteristics by flooring material

**Figure 6:**
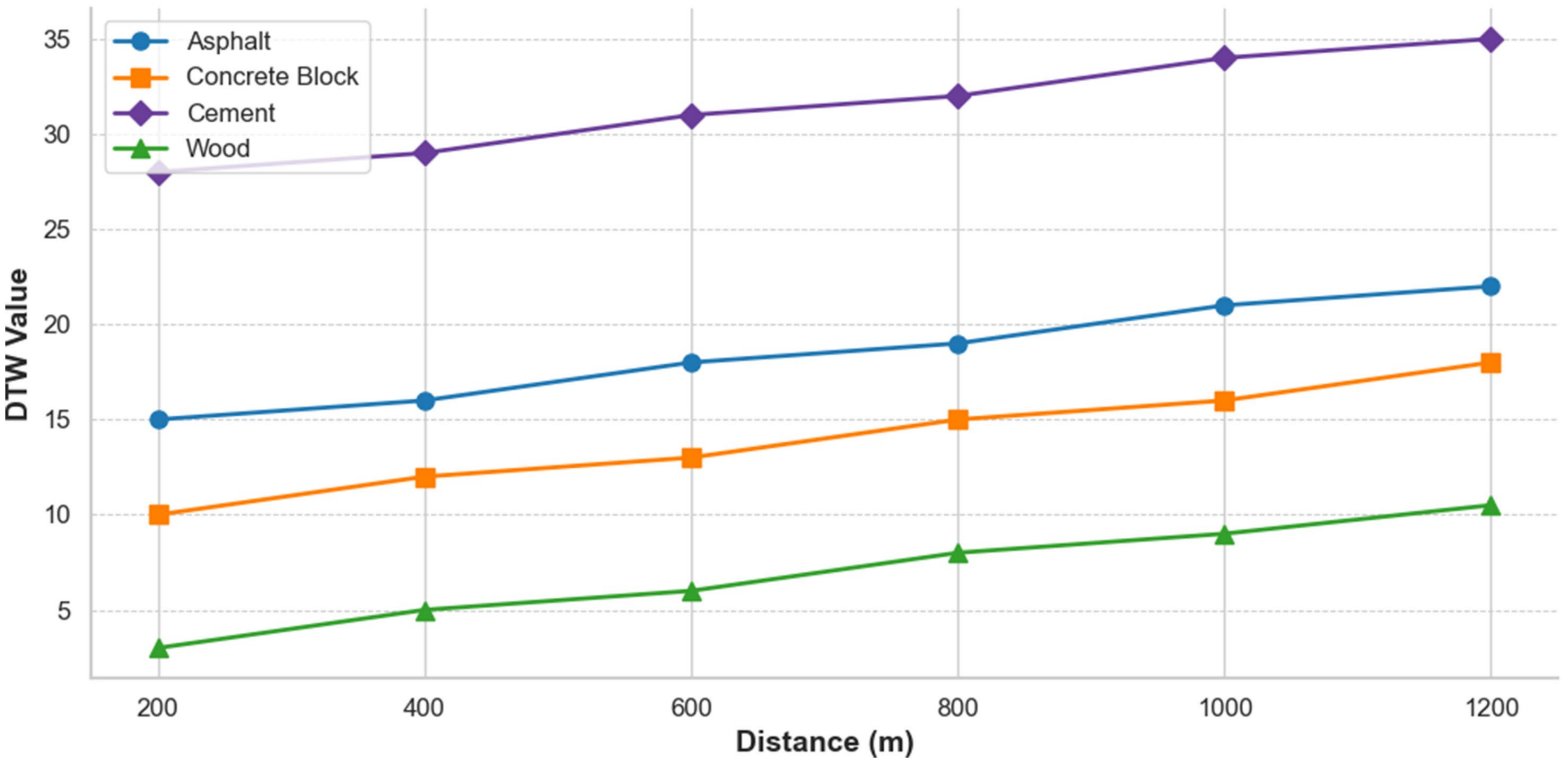
Variation of DTW values for different flooring materials with walking distance.

In this study, DTW values were measured at 200 m intervals from 200 m to 1,200 m for four different flooring materials (cement, asphalt, concrete block, and wood) to analyze their cumulative fatigue and walking characteristics. The analysis showed that DTW values increased with increasing walking distance for all flooring materials, but there were distinct differences in the pattern of increase and absolute value of DTW values for each flooring material. Figure 6 shows the change in DTW values for each flooring material as a function of walking distance from 200 m to 1,200 m.

Cement had the highest DTW mean (39.14), objectively indicating the highest walking fatigue. The specific DTW values were as follows 28.9 at 200 m, 29.3 at 400 m, 31.1 at 600 m, 32.4 at 800 m, 34.1 at 1000 m, and 35.2 at 1200 m. It also had the largest DTW standard deviation (9.74) and range (19.54–70.57), indicating the highest variability in walking patterns. Asphalt had the second highest DTW mean (27.03), indicating a moderate level of walking fatigue. The specific DTW values were as follows 15.1 at 200 m, 15.9 at 400 m, 18.2 at 600 m, 19.1 at 800 m, 21.3 at 1000 m, and 22.1 at 1200 m. The DTW standard deviation (3.43) was relatively small, indicating a consistent walking pattern. Concrete blocks had the third highest DTW mean (24.02), indicating a moderate level of objective walking fatigue. The concrete DTW values were as follows 10.2 at 200 m, 12.1 at 400 m, 13.4 at 600 m, 15.2 at 800 m, 16.1 at 1000 m, and 18.3 at 1200 m. It had the second largest DTW standard deviation (6.06), indicating relatively high variability in gait patterns. Wood had the lowest DTW mean (12.99), objectively indicating the lowest walking fatigue. The specific DTW values were as follows 3.1 at 200 m, 5.2 at 400 m, 6.1 at 600 m, 8.3 at 800 m, 9.2 at 1000 m, and 10.4 at 1200 m. DTW had the smallest standard deviation (3.05) and range (6.59–23.45), indicating the most stable gait pattern.

### Detailed analysis of flooring gait characteristics by age group

**Figure 7:**
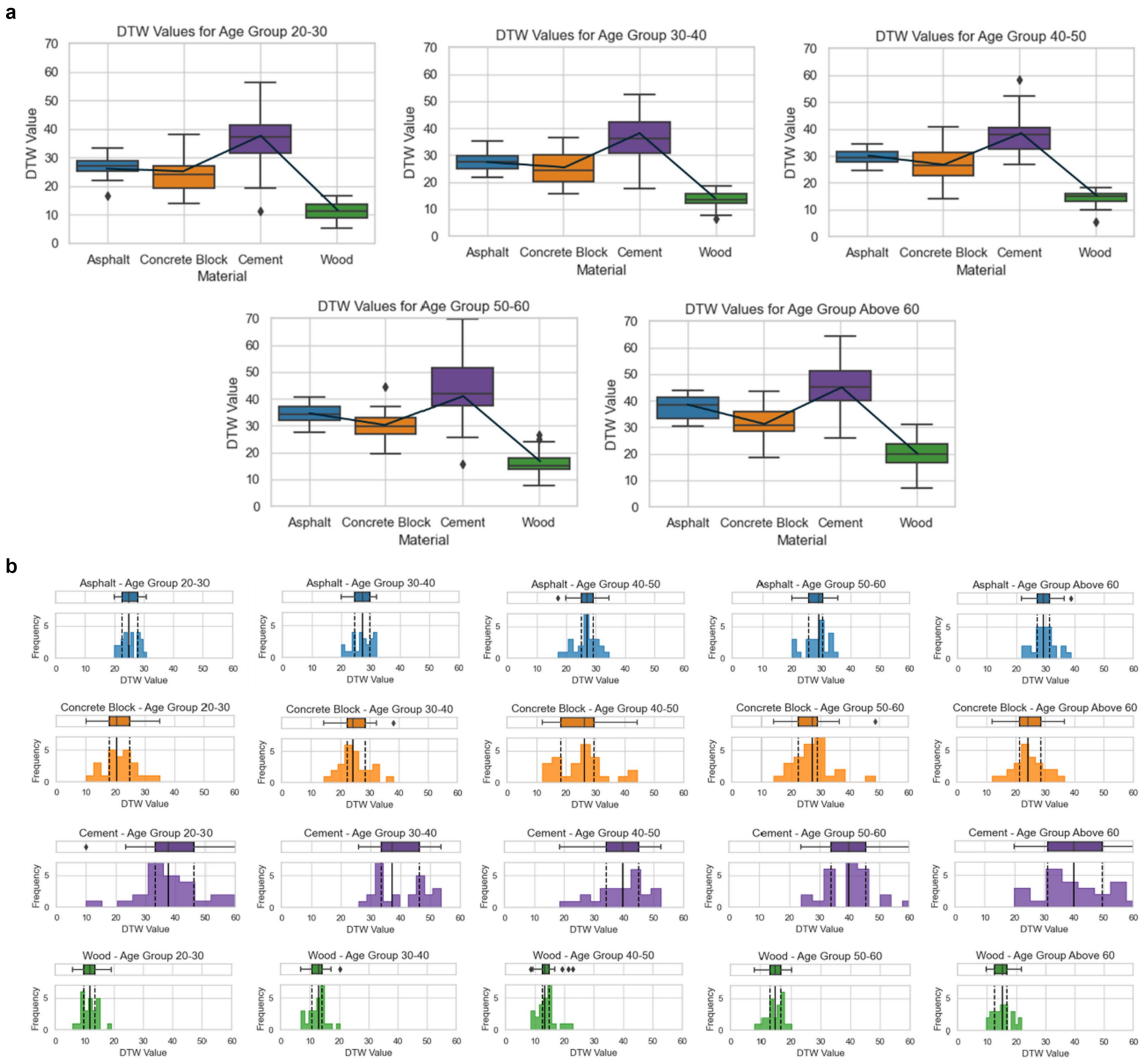
(a) Impact of flooring materials on walking stability across age groups: distribution and trends. (b) Age-related differences in walking stability across various flooring materials: histogram and boxplot analysis.

In this study, we examined the walking characteristics across four flooring materials (asphalt, concrete block, cement, and wood) for five different age groups (20–30, 30–40, 40–50, 50–60, and 60+) using DTW values. To facilitate clarity and detailed interpretation, we have divided Figure 7 into two sections. The first section shows overall DTW values across all age groups for each flooring material, while the second section provides a breakdown of DTW values by age group and flooring type, allowing for a more nuanced understanding of the impact of flooring on different age demographics.

### Section 1: overall DTW value trends across all age groups

Figure 7a displays DTW values across all age groups for each flooring material, enabling a broad comparison across different surfaces. For asphalt, the average DTW value began at 25.48 (SD 3.47) in the 20–30 age group and rose by 15.8% to 29.50 (SD 3.65) in the 60+ age group. Concrete block showed a similar trend, with DTW values increasing 16.8% from 23.12 (SD 6.28) in the 20–30 age group to 27.00 (SD 6.45) in the 60+ group. Cement, which consistently had the highest DTW values, increased by 10.4%, from 37.58 (SD 9.51) in the 20–30 age group to 41.50 (SD 9.70) for those 60 and older. Wood, which maintained the lowest DTW values, exhibited the greatest percentage increase of 31.1%, rising from 11.82 (SD 3.05) in the 20–30 group to 15.50 (SD 3.12) in the 60+ group. When comparing DTW values by flooring material for the 60+ age group, cement had the highest DTW value of 41.50, followed by asphalt at 29.50 (28.9% lower than cement), concrete block at 27.00 (34.9% lower than cement), and wood at 15.50 (62.7% lower than cement). This trend suggests that wood provides the most stable and comfortable walking environment, particularly beneficial for older adult individuals.

### Section 2: age-specific impact of flooring materials on DTW values

Figure 7b breaks down DTW values by both age group and flooring material, providing detailed insights into how different flooring surfaces affect gait stability within each age category. To provide a more detailed breakdown, histograms of DTW values by flooring material were analyzed for each age group. Each flooring material exhibited distinct characteristics in the younger (20–30) age groups. For instance, asphalt DTW values were predominantly distributed in the 20–30 range, with 18 participants clustered around the 25–26 value. Concrete blocks showed a range of 15–35 DTW values, with a concentration of about 12 participants in the 20–25 range. Cement had a broader distribution, spanning 25–55 DTW values, with around 10 participants in the 35–40 range, while wood showed a narrower range of 5–20 DTW values, with the highest frequency at about 22 participants within the 10–15 range. The comparison of DTW values between cement and wood across age groups further highlights the stability provided by wood. For the 20–30 age group, the DTW difference between cement and wood was significant at 25.76 (218.0%), while in the 60+ age group, the difference was still notable at 26.00 (167.7%). This consistent disparity suggests that wood surfaces minimize walking fatigue and enhance stability across all age groups. Additionally, a standard deviation analysis revealed that cement surfaces produced the highest standard deviation (ranging from 9.51 to 9.70 across all age groups), indicating substantial variability in walking patterns on this material. Concrete blocks showed the second-highest variability (SD 6.28–6.45), while wood had the lowest standard deviation (SD 3.05–3.12), demonstrating a consistent walking experience, followed by asphalt (SD 3.47–3.65). These results reinforce that cement may lead to highly variable walking experiences based on individual characteristics, while wood consistently supports stability and comfort. This comprehensive analysis indicates that flooring materials significantly influence walking fatigue and stability, especially with age. While cement floors provide durability, they introduce higher variability and potential instability, particularly for older individuals. Wood surfaces, by contrast, offer a stable and comfortable experience across all age groups, underscoring their suitability for public spaces, particularly in areas frequented by older adult pedestrians.

## Discussion

### Flooring properties, walkability, and implications for urban planning

The analysis of IMU sensor data in this study showed that the physical properties of flooring materials significantly influence walking stability, extending beyond previous research findings. While Li et al. (36) primarily focused on skid resistance properties of different surfaces for walking safety, our results provide quantitative evidence of how material flexibility affects walking stability. These findings support Ferreira et al. (35) observations about the influence of pavement materials on walking patterns, though our study adds quantitative metrics through DTW analysis. The relationship between material properties and walking stability shown in our results aligns with Ren et al. (37) findings on material characteristics, though their focus was primarily on thermal comfort. Our observation that wood provides superior stability (DTW values 12.99) compared to cement (DTW values 39.14) adds new insights to previous studies on material effects. Furthermore, our age-related findings complement Giles-Corti et al. (38) research on environmental factors affecting different age groups, while providing specific quantitative measures of how material effects vary with age. The observed patterns of fatigue accumulation across different materials extend Wong et al. (42) work on pedestrian movement analysis, contributing new understanding of how material properties influence long-term walking stability. The progressive increase in DTW values with distance, particularly notable in harder materials like cement, provides quantitative evidence supporting previous qualitative observations about material-dependent fatigue effects. This shows that material selection is an important factor when designing walkways. In particular, the change in DTW values of different flooring materials with increasing walking distance was not significant at first, but the difference became more pronounced as the distance increased. For example, cement saw a relative increase in DTW values from 28.9 to 35.2 (a 21.8% increase) when going from 200 m to 1,200 m. On the other hand, while Wood showed a smaller absolute starting value, it demonstrated a larger relative increase from 3.1 to 10.4 (a 235.5% increase). However, despite Wood’s larger percentage increase, its absolute DTW value remained significantly lower throughout the walking distance, indicating consistently better walking stability compared to cement. The distinct trend of increasing DTW values over distance varies by flooring material: cement showed a rapid increase of 21.8% (from DTW 28.9 to 35.2) over 1,200 m, while wood demonstrated better stability with consistently lower DTW values despite its percentage increase (from 3.1 to 10.4). These material-specific trends in DTW progression indicate how different flooring materials influence fatigue accumulation during extended walking periods, with harder materials like cement leading to more rapid stability deterioration compared to more elastic materials like wood. The analysis by age group also revealed differences in resilience and adaptability to fatigue. For people in their 20s and 30s, the effect on fatigue was relatively small. This indicates that the same experimental conditions may not have induced enough fatigue in younger people. In contrast, the cumulative effect of fatigue was more pronounced in the older age group. This shows that fatigue accumulation and recovery patterns can differ by age, even when performed by the same person, wearing the same shoes, and in the same environment. The difference in the distribution range of DTW values for each flooring material is also noteworthy. Cement had the widest range, from 19.54 to 70.57. However, wood had the narrowest range, from 6.59 to 23.45. This suggests that cement may cause stronger fatigue depending on individual walking characteristics or environmental conditions, while wood is less fatiguing for most pedestrians. These findings have implications for urban planning, especially for designing walking environments for an aging society. Given the aging trend, materials that provide comfortable and consistent walkability, such as wood, should be prioritized in areas with high concentrations of older people. This can contribute to providing a better walking environment that considers the physical characteristics of the older adult, promoting physical activity and improving their quality of life. Beyond the physical improvement of the walking environment, this approach can lead to increased satisfaction and physical activity among city dwellers.

### Study limitations and future research

The study was analyzed in 80 participants, and the results are significant. The author was able to quantitatively confirm that older people are more susceptible to fatigue and that different flooring materials cause this differently. However, more data is needed to fully validate the patterns found in this study. This would provide stronger support for our findings. Of particular interest is the difference in fatigue induction across age groups. In this experiment, we used the same 1,200 m walking distance for all age groups, which was set at a level that older adults can perform. As a result, participants in their 20s and 30s showed relatively little change in DTW. This suggests that 1,200 m may not have provided enough fatigue for younger participants to observe significant changes. Nevertheless, the differences in fatigue between the different distances and flooring materials were significant. Also, our study on fatigue accumulation provides general insights across all age groups, though limited specific research on fatigue onset by age group may influence the precision of our observations. Future studies should be more multifaceted, using a wider range of distance conditions and flooring materials. For example, for younger participants, the study could be designed to include longer walking distances or higher intensity activities to induce sufficient fatigue. Additionally, future research could involve a more detailed analysis on a single age group to better understand specific age-related fatigue patterns.

A comprehensive fatigue analysis using a combination of sensor data and physiological measurements would allow for a more multidisciplinary approach to data analysis. In this study, this study used IMU sensors instead of EMG (electromyography) sensors. This is because EMG sensors are relatively expensive compared to IMU sensors and can be uncomfortable to wear for long periods of time in a normal walking environment. IMU sensors, on the other hand, are easier to wear, more suitable for long-term use in everyday life, and provide sufficient data to analyze gait patterns. There is also a need to expand the range of participants to better understand the variability of gait patterns across different individual characteristics (weight, height, existing musculoskeletal conditions, etc.). This would provide a deeper understanding of how flooring affects the gait of people with different physical conditions. Furthermore, studies that track participants’ walking patterns over a longer period in their daily lives are needed. This could be accomplished through continuous data collection using wearable devices, which would help to better understand walking patterns and fatigue accumulation in real-life environments.

## Conclusion

Walking is a critical component of urban life, directly influencing the health and quality of life of citizens. Traditional methods for evaluating walking environments have been largely subjective or limited in scope, highlighting the need for more objective and quantitative approaches. This study introduced a novel methodology using IMU sensors and DTW algorithm to evaluate the effects of various flooring materials on walkability across different age groups. Our findings demonstrate that flooring material properties significantly affect walking stability and fatigue accumulation, with wood providing the most stable environment and cement showing the highest instability. The systematic comparison of four common pavement materials revealed clear hierarchical differences in their impact on walking stability, offering valuable insights for material selection in urban design.

Age-based analysis revealed that material effects become more pronounced with age, particularly affecting older adults’ walking stability. This finding is especially significant given global demographic trends toward aging populations, suggesting the need for more thoughtful consideration of flooring materials in areas frequently used by older adult citizens. Additionally, our analysis of walking distance effects provided a new understanding of how different materials influence fatigue accumulation during extended walking periods, with implications for the design of long pedestrian pathways and commercial areas.

These results provide significant implications for urban planning, emphasizing the importance of material selection in creating age-friendly walking environments. The methodology presented here offers a quantitative framework for evaluating walkability that can be applied across different urban contexts and user populations. Furthermore, our findings can serve as a foundation for evidence-based design decisions in developing more inclusive and effective urban walking spaces, particularly important for accommodating aging populations. This research contributes to the broader goal of creating sustainable, accessible, and comfortable urban environments that promote physical activity and enhance quality of life for all citizens.

## Data Availability

The original contributions presented in the study are included in the article/supplementary material, further inquiries can be directed to the corresponding author.
